# Heading recovery from optic flow: comparing performance of humans and computational models

**DOI:** 10.3389/fnbeh.2013.00053

**Published:** 2013-06-21

**Authors:** Andrew J. Foulkes, Simon K. Rushton, Paul A. Warren

**Affiliations:** ^1^School of Psychological Sciences, The University of ManchesterManchester, UK; ^2^School of Psychology, Cardiff UniversityCardiff, UK

**Keywords:** heading, computational models, optic flow

## Abstract

Human observers can perceive their direction of heading with a precision of about a degree. Several computational models of the processes underpinning the perception of heading have been proposed. In the present study we set out to assess which of four candidate models best captured human performance; the four models we selected reflected key differences in terms of approach and methods to modelling optic flow processing to recover movement parameters. We first generated a performance profile for human observers by measuring how performance changed as we systematically manipulated both the quantity (number of dots in the stimulus per frame) and quality (amount of 2D directional noise) of the flow field information. We then generated comparable performance profiles for the four candidate models. Models varied markedly in terms of both their performance and similarity to human data. To formally assess the match between the models and human performance we regressed the output of each of the four models against human performance data. We were able to rule out two models that produced very different performance profiles to human observers. The remaining two shared some similarities with human performance profiles in terms of the magnitude and pattern of thresholds. However none of the models tested could capture all aspect of the human data.

## Introduction

**Figure 1:**
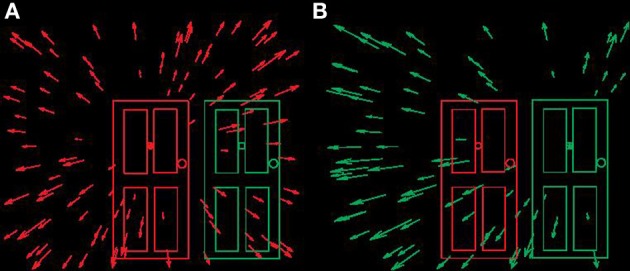
**Schematic optic flow fields available at the eye when locomoting towards doorways. (A)** Observer locomoting towards left “no entry” doorway. **(B)** Observer locomoting towards right “entrance” doorway. In both panels direction of heading (instantaneous direction of locomotion) indicated by the centre (“focus of expansion”) of the radial flow field.

Optic flow is the pattern of optical motion available at the eye during relative movement between the observer and the scene. It is known that primates are sensitive to the stereotypical patterns of optic flow which arise when an observer moves through a largely stationary scene (Figure 1). This sensitivity has been demonstrated using psychophysics (e.g., Warren and Hannon, 1988; Snowden and Milne, 1997), neurophysiology (e.g., Duffy and Wurtz, 1991; Wurtz, 1998) and neural imaging (e.g., Morrone et al., 2000; Smith et al., 2006). See Lappe et al. (1999) for a review.

Several roles for the neural processing of optic flow have been put forward. For example, Lee and Aronson (1974) found that movement of an artificial room around a stationary observer could cause infants to sway, and in extreme cases fall over, providing strong evidence that optic flow plays a role in the control of posture. An additional role proposed suggests that that optic flow drives rapid eye movements which act to stabilize the foveal image and maintain correspondence between images on the two retinae (e.g., see Busettini et al., 1997). Consequently, this optic flow-driven stabilization process helps to preserve foveal visual acuity and stereo vision during observer movement (Angelaki and Hess, 2005). More recently, in the *flow parsing hypothesis*, it has been suggested that optic flow processing plays an important role in the assessment of scene-relative object movement during self movement (Rushton and Warren, 2005; Rushton et al., 2007; Warren and Rushton, 2007, 2008, 2009a,b; Matsumiya and Ando, 2009; Pauwels et al., 2010; Warren et al., 2012).

Although many roles for optic flow processing have been suggested, the vast majority of work in the literature to date has focused on a single potential role in the guidance of locomotion. Following Grindley (see Mollon, 1997) and contemporaneously with Calvert (1950), Gibson (1950) championed the idea that the primary role of optic flow is used in the guidance of locomotion (however see Llewellyn, 1971; Rushton et al., 1998 for alternative hypotheses). During forward movement of the observer a radial pattern of optic motion is available at the eye. The centre of the radial pattern, termed the Focus of Expansion (FoE) coincides with the direction of locomotion of the observer relative to the environment. For example, if the FoE is coincident with a doorway then an observer regulating direction of locomotion to maintain the FoE in the same location will reach the doorway (see Figure 1). In a seminal publication by W. Warren (Warren and Hannon, 1988) it was demonstrated that stationary observers can judge simulated direction of locomotion with a precision of a degree or two based only on optic flow information consistent with self movement.

A number of models of the computations underlying heading estimation (or more generally methods for estimating eye or camera movement parameters from sequences of static images) have been proposed (e.g., Longuet-Higgins and Prazdny, 1980; Heeger and Jepson, 1992; Perrone, 1992; Lappe and Rauschecker, 1993; Royden, 1997; Beardsley and Vaina, 1998; Beintema and van den Berg, 1998; Fitzgibbon and Zisserman, 1998; Grossberg et al., 1999; Wang and Cutting, 1999; Davison, 2003). These models vary markedly in terms of approach and aims. For example a set of models (primarily published in the computational vision literature) aim to estimate parameters of camera movement as accurately as possible from the flow field (or, equivalently, sequences of static images). In these cases a novel mathematical treatment of the information available in the flow is commonly the primary inspiration for the model (e.g., Longuet-Higgins and Prazdny, 1980; Heeger and Jepson, 1992; Fitzgibbon and Zisserman, 1998; Davison, 2003). For several other models (most commonly published in the human vision or biological literature), the aim is to model human performance in heading estimation, including circumstances in which performance is poor. These models are often more clearly inspired by known properties of the neural substrates thought to underpin heading estimation (e.g., Perrone, 1992; Beintema and van den Berg, 1998). Further models have, at least partially, reconciled computer vision models with what is known about human motion processing (e.g., Royden, 1997 for Longuet-Higgins and Prazdny, 1980; Lappe and Rauschecker, 1993 for Heeger and Jepson, 1992). We might also add a further class of model which uses a connectionist approach (e.g., Beardsley and Vaina, 1998; Grossberg et al., 1999); this class is clearly related to the categories above in that it seeks to reflect neurophysiological constraints, however it is distinct in that the known neurophysiological constraints emerge as properties of the connectionist network rather than being used at the outset to underpin the architecture of the model. Lastly we note that although the majority of models take the instantaneous monocular 2D vector flow field generated from combinations of rotations and translations of the eye, it has been shown that humans can take advantage of stereo depth information (van den Berg and Brenner, 1994; Rushton et al., 1999) and some models take stereoscopic flow fields as their input (e.g., Wang and Duncan, 1996). For a thorough treatment of different classes of model for heading estimation see Lappe (2000), and see Raudies and Neumann (2012), for a recent review and evaluation.

Here we investigate how well candidate models of heading perception capture human performance. We first assess how the performance of human observers varies as a function of the quantity (i.e., number of flow dots per frame) and quality (i.e., amount of 2D directional noise in the flow field) of motion information in the flow field. This provides a reference performance profile. We then select four models that span the approaches to modelling heading estimation and compare their performance to that of human observers; we generate similar performance profiles for our candidate models and compare them to the reference human profile.

The quality and quantity manipulations are motivated by the fact that models of heading recovery differ markedly in their treatment of the motion information in the flow field. While some implement inherently local operations, such as motion vector differencing (Longuet-Higgins and Prazdny, 1980), others involve global processing such as template matching flow over the entire field of view (Perrone, 1992). Consequently, manipulating the quantity and quality of the information in the flow field should have differential effects on the models.

In the first section we assess how human performance is affected by the quality and quantity of information in the flow field. In the second section we present the same stimuli to four heading models. The performance profiles of the four models are then compared to human performance.

## Experiment—human observers

### Methods

#### Participants

Nineteen participants (6 male, 13 female) took part in the experiment. All were recruited from the School of Psychological Sciences, University of Manchester. Two of the participants were authors (AJF & PAW), and the other 17 (2 Postdoctoral Research Associates, 5 Ph.D. students, and 10 undergraduate students) were naïve. All participants were recruited and tested in line with the Declaration of Helsinki. The study was approved by the ethics committee in the School of Psychological Sciences, University of Manchester.

#### Apparatus

Participants sat in a dark room with their chin on a chinrest such that their line of sight was directed at the centre of a CRT display (Viewsonic pf255) running at 100 Hz with a resolution of 1280 × 1024 and subtending a visual angle of approximately 40° × 32°. The display was approximately 57 cm from the observer's eyes. The visible part of the display casing was obscured with irregular shaped black card, which minimized stray light reflecting on the casing.

The stimuli were generated under Windows 7 on a Dell Optiplex 780 with an nVidia GeForce 9600GT using x16 anti-aliasing.

The experiments were written using Lazarus, a free open source development system for Pascal (http://www.lazarus.freepascal.org/) together with the JediSDL libraries (http://pascalgamedevelopment.com/jedi-sdl/) which facilitate use of OpenGL in Pascal.

#### Stimuli and task

**Figure 2:**
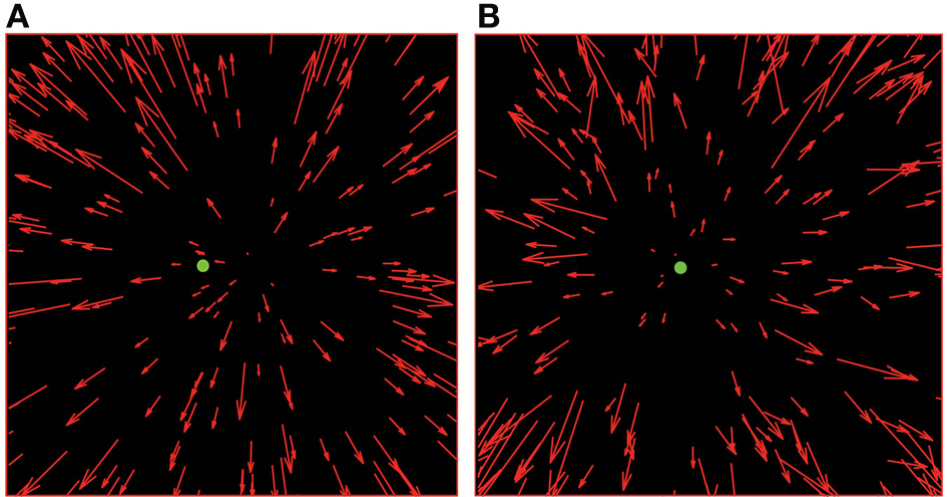
**Schematic illustration of radial optic flow fields presented in the experiments**. In both panels, there are 200 dots, with the target/fixation point located at 2° to the left of the centre of the field of view. In **(A)**, the FoE is located 4° to the right of the target position and there is no noise present. In **(B)**, the FoE is located 0.2° to the left of the target position and there is directional noise present (15° s.d.—Noise Level 2).

Each trial consisted of three parts. The first was the *fixation phase*, in which the target line (a 1° vertical red line placed at one of four target locations) alone was presented for 1 s and participants were instructed to fixate it. In the second part, the *flow phase*, the target line turned into a small green annulus with outer radius of 0.1° and inner radius of 0.05°. While the observers maintained fixation the flow field was presented on the screen for 2 s. Flow fields were comprised of limited lifetime (250 ms) red dots, each of 0.1° radius. The dots moved in an expanding radial pattern consistent with forwards translation of the observer at around 1 m/s through a cloud of dots at distances randomly assigned in a range between 0.5 and 4.5 m from the observer (see Figure 2A).

The third and final part of the trial was the *response phase* during which observers were once again presented with the red target line (same as in the fixation phase) in isolation, which remained onscreen until the observer made a response. The observer's task was to indicate whether they were passing to the left or right of the target line.

#### Choice of flow fields

We made a decision to only test performance for simple radial flow fields. This decision was made for several reasons. First, the candidate models were only designed for the case of forward translation of the observer. Second, it makes logical sense to start with the most basic optic flow stimulus. If model performance is markedly different in the simplest case then there is no need to consider it any further as a candidate. We did not consider the effects of adding simulated gaze rotation during forward translation. Although, historically there has been much interest in such flow fields because of the debate regarding the role of extra-retinal information in heading recovery (e.g., Warren and Hannon, 1988; Royden et al., 1992), today these issues have been largely resolved (e.g., see Li and Warren, 2002).

#### Design

The experimental design was based on that of Warren et al. (1988). We manipulated two variables, the quantity (number of dots—5, 50, 100, 200 dots per frame) and quality [dot noise—a random independent additive perturbation of the 2D direction of each flow field dot by sampling from a zero-mean Gaussian distribution with standard deviations set at either 0° (no noise), or 7.5° (noise level 1), or 15° (noise level 2)] (see Figure 2B). Two other variables were systematically varied, these were target location (±2°, ±4°) and FoE offset relative to the target location (±0.2°, ±0.5°, ±1°, ±2°, ±4°). It should be noted that given the limited lifetime of our dots (250 ms) and an estimate of visual persistence of around 100 ms (see Di Lollo, 1980) we expect that the observers perceive approximately 40% more points than are present on any single frame.

Full factorial combination of the variables resulted in 480 individual conditions and observers saw each condition twice over two 30 min experimental sessions. For the data analysis, similar to Warren et al. (1988) we collapsed the data over the positive and negative FoE offsets and then over target locations, giving 16 repetitions for each of 5 (FoE offsets) × 4 (number of dots) × 3 (dot noise) = 60 conditions.

#### Analysis

Analysis of the data was based on that in Warren et al. (1988). For each of the 12 (number of dots × dot noise) flow field conditions, participant responses were converted to % correct scores and cumulative Gaussian psychometric functions were fitted to these scores as a function of the FoE offset. Thresholds were defined as the FoE offset for which participants performed at the 75% correct level.

In order to minimize the impact of individual differences between participants, thresholds were normalized within individual. The normalization procedure involved first calculating the average threshold for each observer over the 12 flow field conditions (the grand mean), and then dividing each threshold by the grand mean.

When averaging over observer data we also took into account the quality of the psychometric function fit to each participant's heading response data. Specifically, the mean normalized threshold for each of the twelve conditions was calculated as a weighted linear sum of the individual participant thresholds. Weights were defined based on the inverse of the RMS error in the psychometric function fit, normalized to sum to 1 (see Appendix for details). Finally we converted the normalized data back to an angular measure by multiplying it by the grand mean of thresholds calculated over all participants.

### Results—humans

**Figure 3:**
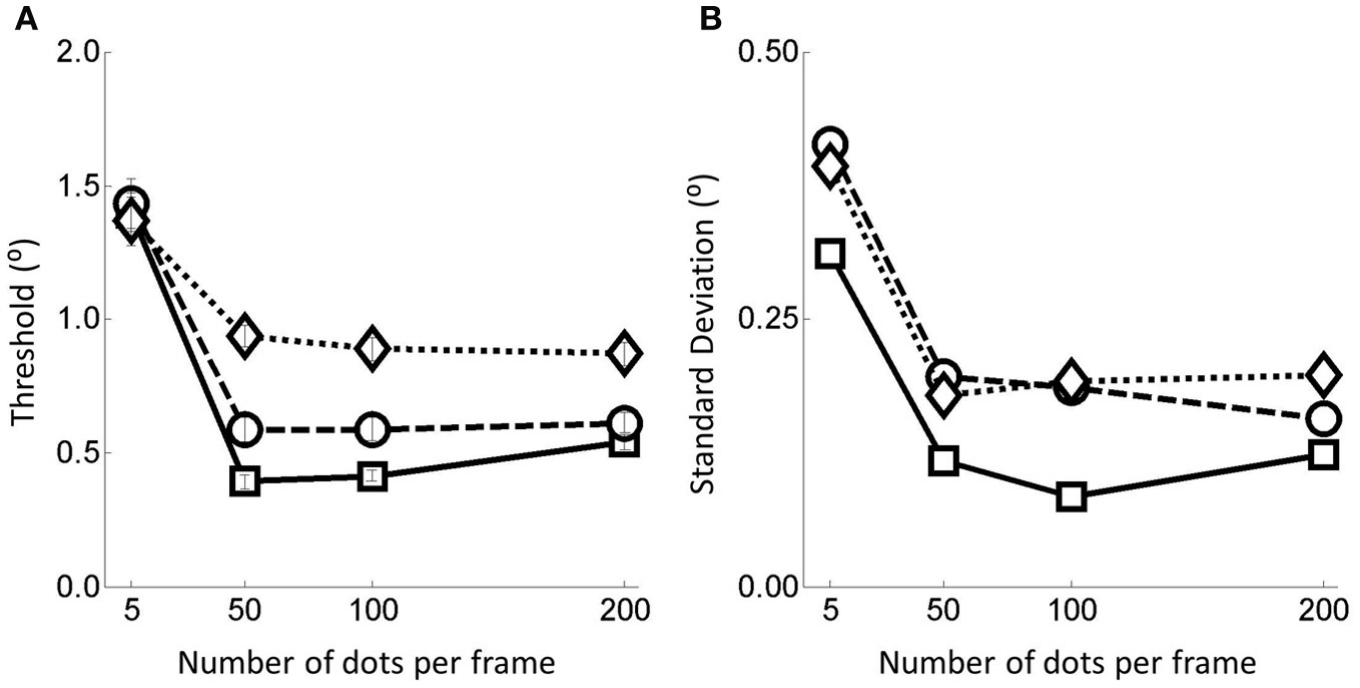
**(A)** Thresholds and **(B)** associated standard deviations for the 12 flow field conditions. Error bars represent ±1 s.e. Squares represent no noise conditions, circles for noise level 1 and diamonds for noise level 2.

Weighted thresholds for the twelve flow field conditions are shown in the Figure 3A. The magnitude of the thresholds is consistent with those found in previous studies (e.g., Warren et al., 1988).

Considering the number of dots per frame manipulation first, we see that performance is markedly worse below 50 dots per frame but that above this level performance is relatively constant. This finding was supported by statistical analyses in the form of multiple weighted *t*-tests (Bland and Kerry, 1998) conducted within each noise condition revealing highly significant differences between the thresholds in the 5 and 50 dots per frame conditions for all three noise conditions but no differences between thresholds in the other dots per frame conditions (see Table 1).

**Table 1:** **Results of weighted *t*-tests conducted within noise condition**.

	**No noise**	**Noise level 1**	**Noise level 2**
(5,50)	0.0040^*^	0.0081^*^	0.0024^*^
(50,100)	0.3132	0.6844	0.5065
(100,200)	0.7453	0.4718	0.7274

For each pair of sequential number of dots per frame levels, p-values are reported at the noise level given in the column heading. Asterisks indicate that the fit was significant.

Turning to the noise manipulation we see that there is a clear effect of noise such that increasing the noise level leads to increased thresholds. Beyond the 50 dots condition the effect of moving from the no noise condition to noise level 2 is an approximate doubling in threshold (from around 0.5° to around 1.0° on average). For the 5 dots condition, the effect of noise is much smaller, with little or no change in the threshold measured. These observations are supported by weighted *t*-tests (Bland and Kerry, 1998) conducted within number of dots per frame conditions revealing significant differences between the no noise and noise level 2 conditions in all dots conditions except for when there were 5 dots per frame (Table 2).

**Table 2:** **Results of weighted *t*-tests conducted within number of dots per frame condition**.

	**5**	**50**	**100**	**200**
NN, NL1	0.3135	0.0019^*^	0.0230^*^	0.3481
NN, NL2	0.4004	<0.0001^*^	0.0034^*^	0.0090^*^
NL1, NL2	0.9110	0.0414^*^	<0.0001^*^	0.0402^*^

For each pair of noise levels, p-values are reported at the number of dots per frame level given in the column heading. Asterisks indicate that the fit was significant.

For later comparison with the heading models we also plot the within subject weighted standard deviations of the human data as a function of the manipulations in Figure 3B. Primarily, the human s.d.s follow the pattern of the thresholds, increasing when there is either more noise or there are fewer dots in the field.

**Figure 4:**
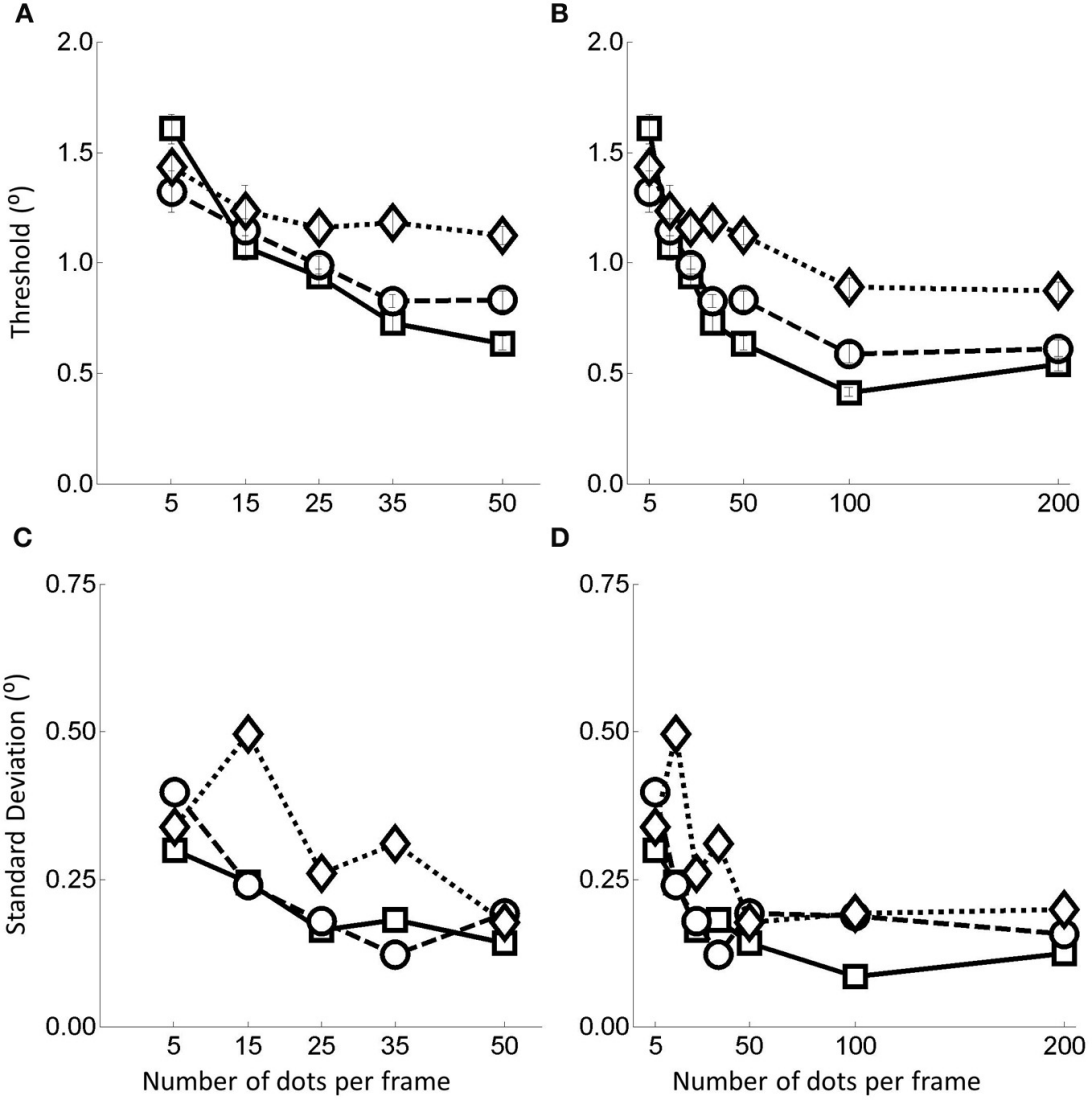
**(A)** Thresholds plots for the follow up experiment for the dot range 5–50. **(B)** combined data, **(C)** standard deviation for the 5–50 data, and **(D)** standard deviation for the combined data. In all cases, the squares represent no noise, circles noise level 1, and diamonds noise level 2. Error bars represent ±1 standard error.

Because the most significant change in performance occurs between 5 and 50 dots we conducted a follow up experiment to explore this range in more detail. The second experiment was identical to the first except that the number of dots per frame levels were set as (5, 15, 25, 35, 50). The results for this experiment with 20 additional participants (three of whom also took part in the first experiment) are shown in Figure 4A. Note that the data presented in the 5 and 50 dots per frame conditions were obtained by collating data from the 36 different participants taking part in these two conditions in both the primary and follow up experiments). In Figure 4B we re-plot the data from the primary experiment with the data in Figure 4A inserted (note again that the data at 5 and 50 dots per frame are similar to those in Figure 3A, i.e., they have been calculated over the 36 participants who saw these two conditions over the two experiments). Again, for later comparison with the models, in Figures 4C,D we also show the within subjects weighted standard deviations of the human data as a function of the flow quality and quantity manipulations.

The data in Figure 4A suggest that, similar to the data in the primary experiment the thresholds decrease and stabilize as the quantity of flow information increases and that the stabilization begins to occur somewhere in the 15–35 dots per frame region.

It is worth noting that in Figure 4B there appears to be a further drop in thresholds between 50 and 100 dots. This contradicts the pattern of data found in the primary experiment in which performance had stabilized by 50 dots per frame. We think that this is at least partially due to a mismatch in thresholds between the preliminary and follow up experiments in the 50 dots per frame condition such that those in the follow up experiment were rather larger than in the main experiment. Consequently the drop in thresholds observed in this range may simply be a consequence of collecting the data in two separate experiments rather than reflecting some residual improvement in performance in that range.

## Discussion—human data

By manipulating the quantity (number of dots per frame) and quality (amount of directional noise) of information in the optic flow field we generated a performance profile for human observers. From the performance profile we note the following features for evaluating the four candidate heading models.

As the number of dots per frame is increased a critical range is reached [between 15 and 35 dots per frame—see Warren et al. (1988) who report similar threshold values]. Before reaching that range heading thresholds exhibit a marked dependence on the quantity of flow in the field such that as the quantity is reduced the thresholds increase rapidly. After that range as the quantity is increased thresholds remain relatively stable. Last, before reaching the critical range there is little evidence of a relationship between heading performance and the amount of directional noise in the flow field whereas afterwards there is a marked relationship between heading performance and directional noise (as noise increases so do the thresholds).

In the following sections we describe a number of heading models and assess their compatibility with these features.

## Heading models

We begin with a brief description of the models tested. In the interests of brevity the descriptions presented here are deliberately concise, further details can be found in the Appendix together with simulations which demonstrate our implementations reproduce the behavior of the original models.

### Candidate models

We have chosen four heading models which were first described in: (1) Longuet-Higgins and Prazdny (1980); (2) Perrone (1992); (3) Heeger and Jepson (1992); and (4) Wang and Cutting (1999). The models will be referred to as LHP80, P92, HJ92, WC99, respectively. In all cases the input to the model was a 2D optic flow field.

#### Longuet-Higgins and Prazdny (1980) (LHP80)

The motivation for LHP80 was primarily computational, i.e., it is based on a mathematical treatment of the available information in order to recover camera movement parameters as accurately as possible. Royden (1997) proposed a biological implementation of this algorithm using properties of MT-like cells.

This model is designed to recover both the translational and rotational velocities of the observer (camera) as it moves through the scene, LHP80 searches for the FoE, (referred to as the vanishing point in LHP80). In order to recover the FoE, the model takes advantage of the fact that for two points in the optic flow field at the same location in the image plane but at different depths in the scene, the rotational components of the flow field are identical but the translational components differ. The model applies a local differencing operation on the velocities of pairs of points in the optic flow field at the same location in the image plane but at different depths in the scene. As a consequence, the FoE can be recovered relatively simply using basic geometry provided sufficiently many pairs (2) of coincident points in the image plane can be identified. Once the FoE has been located, the rest of the unknowns, i.e., the 3 translational and (if present) 3 rotational velocity components can be determined. See the Appendix for more details.

One pertinent issue for LHP80 is that it is assumed that there are at least 2 pairs of points which are co-incident in the image plane but at different depths in the scene. As a consequence this model might be expected to be sensitive to changes in the quantity of information in the flow field, i.e., it will make large errors in heading estimation when there are no (or not sufficiently many) co-incident points. Given the nature of the stimuli in the present experiment (random dot flow fields) it is possible that there will be no co-incident points present in the stimulus. Consequently, we implement a modified LHP80 which after a pair-wise comparison of points in the field selects those which are closest together in the image plane for use in the calculation of heading.

A further issue for this model is that the analysis undertaken relies fundamentally on a local analysis of the velocity information (since it involves a local differencing operation). Consequently, it is possible that this model will suffer when noise is added to the flow field since it does not benefit from an integrative approach which can improve robustness to noise. However, the model could be improved by weighting and combining information across many pairs of points.

Although LHP80 was not tested explicitly in Longuet-Higgins and Prazdny (1980), in the Appendix we demonstrate how accurate this model can be given appropriate velocity information.

#### Perrone (1992) (P92)

This model is based directly upon extensively studied (e.g., Zeki, 1980; Van Essen et al., 1981; Maunsell and Van Essen, 1983; Albright, 1984, 1989) properties of cells in MT and consequently it is biologically-inspired. The fundamental units of the model are local (i.e., relatively small receptive field) speed and direction tuned cells in MT. From these basic units more complex global (i.e., large receptive field) motion processing sensors (or templates) are created by pooling activity from appropriately tuned local units. Crucially, different combinations of local units at different locations in the visual field can be constructed to generate sensors tuned to different heading directions. It is suggested that these sensors reflect the properties of cells in MST in being tuned for global optic flow commensurate with observer translation. This is essentially a template-matching model with the global motion sensor (or template) which is most active taken to signal the current observer heading.

We initially used templates tuned for heading directions over a 40 × 40° grid separated by 1° in the vertical and horizontal extents. This was done to match the notional resolution of other models implemented (see below).

In Perrone (1992), several tests of model performance were carried out. In the Appendix we replicate one of these tests.

#### Heeger and Jepson (1992) (HJ92)

Similar to LHP80, Heeger and Jepson (1992) present a computational treatment of the problem of recovering camera movement (and scene structure) from optic flow information (although they do discuss how the algorithm might be implemented neurally). More, recently Lappe and Rauschecker (1993) have suggested that such a mechanism might be implemented in human MST. HJ92 uses what the authors refer to as a *subspace method* which involves re-expression of the equations of optic flow as a product between two matrices. The first matrix is dependent on the unknown translation component and a second matrix contains the unknown rotation and depth components. The authors then present a method for recovering a least-squares estimate of the translation component. This method involves the definition of a residual function which measures the extent to which a candidate translation is consistent with the flow vectors in the image. The candidate for which the consistency is maximized is taken as the best estimate of translation and can be used to subsequently estimate the rotation and depth components of the scene. A more thorough description including the mathematical formulation is presented in Appendix B.

Heeger and Jepson (1992) tested the robustness of their model to noise and variations in the amount of motion information in the display. In the Appendix, we show that we can faithfully replicating those results. We also note that in the testing of their model the resolution for candidate headings was approximately 1°. We used a similar 1° resolution in our implementation.

#### Wang and Cutting (1999) (WC99)

This model stands out from the others presented thus far in that it is probabilistic in nature, i.e., it returns a distribution on possible heading directions defining the probability that each heading direction generated the flow field. Probabilistic models of perception are very common in the broader literature [e.g., for a review see Rao et al. (2002)] and consequently we felt it was important to include an example. The success of the model is based on a theorem which states that for any given heading direction if a pair of image points are converging then the heading direction cannot be between those two points (Wang and Cutting, 1999). Using this theorem the authors present a Bayesian analysis of heading estimation which looks at the angular velocities of pairs of points in the image to assess whether they are converging or diverging. By considering many pairs of points it is possible to build up a posterior distribution on the heading direction.

The method used in this model is very simple from a computational point of view. However Wang and Cutting (1999) demonstrate that it performs relatively well assuming the flow field is not too sparse. As with the other candidate models, in the Appendix we present the results of simulations for comparison to sample results published in Wang and Cutting (1999). We also note that in the testing of their model, Wang and Cutting split the visual field into columns with a width of 1°, and we use this resolution in our implementation of the model.

### Further information on heading models

Above we briefly describe four candidate models. Details of the implementations can be found in the Appendix. We also refer the interested reader to Lappe (2000) and a more recent and very thorough paper by Raudies and Neumann (2012) comparing the performance of 13 computational models of heading recovery (including variants of those described here). Raudies and Neumann (2012) rigorously characterize and evaluate model performance for a range of manipulations. Similarly to our study, the authors tested model sensitivity to noise and density manipulations. However, they also considered (among other things) simulated ego-motion type, noise model, robustness to statistical bias and depth range.

### Methods

The experimental methods for the model observers were very similar to those for human observers. However, some minor modifications were undertaken to simplify the implementation.

First, the target location was fixed at the centre of the image such that FoE of the flow field was always offset to the side of the central target location. In the human judgment experiments we varied the position of the FoE to minimize any effects associated with repetitive presentation of the same stimuli. Such a manipulation is clearly unnecessary for the models.

Second, since the model cannot “see” the stimulus for 2 s we attempted to achieve parity between flow information provided in trials for human and model observers. It has been estimated that heading judgments require approximately 270 ms (van den Berg, 1992) of optic flow. Over a 2 s trial this would equate to approximately seven independent heading estimates. Consequently on a single trial for the simulated observers, the model returned the average heading estimate obtained over seven independent estimates, each based on two frame (separated by 270 ms) flow-fields.

Third, we needed to take into account the temporal integration window that characterizes human perception. As noted in the methods above, it is estimated that motion perception involves integrating information over 100 ms (Di Lollo, 1980). Given the limited lifetime of our dots (250 ms) we estimate human observers had access to 40% more dots at any given moment. Consequently in each number of dots per frame condition we provided the heading models with 40% extra dots.

Finally, in order to get an accurate estimate of each model's performance profile we ran the models in each condition until the standard error of the mean for that condition dropped below a threshold value of 0.2°. In addition, we were also keen to investigate whether the profile of variability in the different conditions matched that of humans. Consequently we calculated the standard deviation over 1000 trials for each condition for comparison with human observers' precision data.

### Analysis

The analyses undertaken for the model observers are similar to those for humans. We took the data for each model, fitted a psychometric curve (as described in the section above), and calculated the 75% threshold. The weighted (based on the goodness of fit of the psychometric function) means of these thresholds were then used to compare overall model performance.

### Results—models

**Figure 5:**
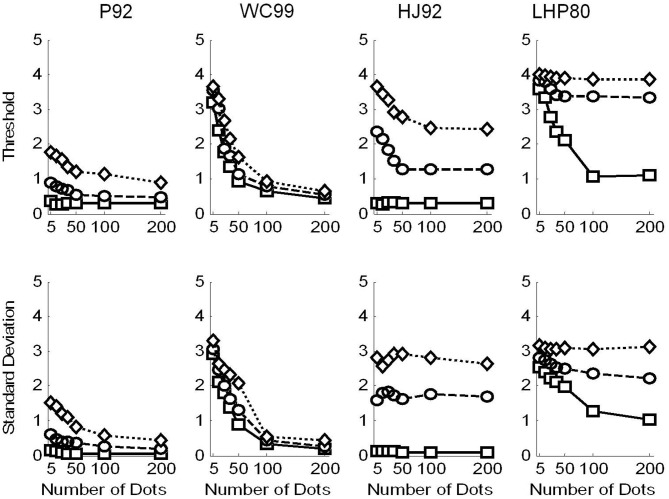
**Raw data for the model observers**. Squares are for no noise, circles for noise level and 1 and diamonds noise level 2. Top row shows the thresholds recovered in each condition. Simulations terminated when sufficient replications had been conducted to drive the standard error of the mean below 0.2°. Bottom row shows the variability (SD) in the model estimates over 1000 repetitions.

In Figure 5 we show the raw threshold data for the different models. Clearly the models perform differently in response to the manipulations of flow quality and quantity.

Considering the thresholds first (top row in Figure 5), we see that all models exhibit sensitivity to noise—as noise is increased the thresholds tend to increase. However, WC99 appears to be rather more robust than any other model to the noise in the flow field.

In addition, all models except for the LHP80 model in the noisy conditions and the P92 and HJ92 models in the no noise condition, exhibit a characteristic pattern of dependence on the number of dots per frame such that as the quantity of flow increases the thresholds decrease up to a point at which the performance reaches a ceiling. In contrast, for LHP80 although thresholds do appear to depend on the quantity of flow when the stimulus contains no noise however, this dependence is markedly reduced when noise is added to the stimulus. We note also that WC99 appears to differ from the other models in the sense that performance continues to improve beyond the 100 dots per frame level. In contrast, for all other models the performance is stable by around 50 dots per frame (or below this point in the no noise condition).

Turning to the variability data (bottom row of Figure 5) we see that the standard deviations appear to largely follow the patterns of the thresholds. However this is not the case for the HJ92 model for which variability is independent from the flow quantity manipulation for all levels of the flow quality factor.

## Human vs. models comparison

A preliminary comparison of the model and human data suggests that none of the models provides a perfect fit to the data. In some conditions human observers outperform the models (e.g., in the noise level 2 conditions). In other conditions certain models outperform the humans (e.g., P92 and HJ92 in the no noise conditions).

We now discuss similarities and differences between models and the human data for each model in turn.

### P92

The pattern of data obtained from this model exhibits some qualitative similarities to that obtained from human observers. Thresholds show sensitivity to the flow quality and flow quantity manipulations. In addition, the magnitudes of the P92 simulated thresholds appear closer than any other model to those seen in human observers. However, P92 thresholds appear to be too low when there is no noise but there are few dots in the field suggesting that this model can cope considerably better than humans in sparse flow fields when there is no noise.

### WC99

This model also demonstrates some qualitative similarities to the human data. In particular it shows sensitivity to both the quantity and quality manipulations. However, for this model, thresholds are considerably higher than those for humans. This is particularly evident in the lowest flow quantity conditions but even at 50 dots per frame thresholds are higher than those seen for humans. In addition, whilst the performance of human observers appears stabilize as the flow quantity increases (and based on the data in Figure 3A we suggest that stabilization occurs at around 15–35 dots per frame), WC99 thresholds still improve at 200 dots per frame relative to 100 dots per frame.

### HJ92

The pattern of thresholds exhibited by HJ92 is similar to those of P92 but considerably higher when noise is present in the flow field. Consequently although a similar dependence on flow quality and quantity is observed, human performance is rather more robust to noisy flow fields. In contrast HJ92 performs better than humans when there is no noise present and, similar to P92, does not show a large increase in threshold when the flow field is noise-free but sparse.

### LHP80

Of the four models tested LHP80 appears to fit the human data least well. First, thresholds recovered by this model are considerably higher than those from humans. In addition, although there is some dependence on flow quantity when there is no noise present, unlike humans LHP80 still improves as the quantity of flow increases from 50 to 100 dots per frame. In addition, it appears that the model cannot tolerate the addition of noise to the flow field. In comparison human observers appear relatively robust to this manipulation.

### Regression analyses

In order to compare the performance profile of each model to the human data more formally, we regressed each of the model thresholds, *t*_*M*_, and standard deviations, *s*_*M*_, against the human data (*t*_*H*_, *s*_*H*_) using simple linear models of the form:

$$\begin{array}{l} t_{H}=\alpha +\beta t_{M}\\ s_{H}=\alpha +\beta s_{M} \end{array}$$

We were particularly interested in the quality of the fits (as measured by the *R*^2^ statistic). We can think of this statistic as telling us about whether the human and model data have a similar pattern or shape. For a model which provided a good approximation to the human data we would expect fits to both thresholds and standard deviations to produce high *R*^2^ values. We were also interested in the value of the slope parameter β for the threshold fits. This parameter reflects the gain of the model with respect to the human data and should be close to 1 if model and human thresholds are similar in magnitude.

Primarily we were interested in the fits to all the data obtained across both experiments (data in Figures 4B,D—referred to as “All” data) but for completeness we also present the fits to the separate data obtained in the primary experiment (data in Figure 3—referred to as “5–200” data) and the follow up experiment (data in Figures 4A,C—referred to as “5–50” data).

The results of the regression analyses are shown in Tables 3 and 4. Table 3 shows the *R*^2^ statistic obtained for each model when regressing both thresholds and standard deviations to the human data. Table 4 show the associated values of the gain parameter β.

**Table 3:** ***R*^2^ statistic for each model for both the thresholds and the standard deviations**.

	**Threshold**			**Standard deviation**		
	**5–50**	**5–200**	**All**	**5–50**	**5–200**	**All**
P92	0.239	0.357^*^	0.315^*^	0.418^*^	0.323	0.427^*^
WC99	0.779^*^	0.827^*^	0.814^*^	0.363^*^	0.748^*^	0.419^*^
HJ92	0.212	0.320	0.274^*^	0.143	0.025	0.086
LHP80	0.561^*^	0.447^*^	0.506^*^	0.119	0.019	0.061

Asterisks indicate that the fit was significant.

**Table 4:** **Gain parameters (β) for each model for both the thresholds and the standard deviations**.

	**Threshold**			**Standard deviation**		
	**5–50**	**5–200**	**All**	**5–50**	**5–200**	**All**
P92	0.240	0.489^*^	0.352^*^	0.134^*^	0.136	0.140^*^
WC99	0.259^*^	0.283^*^	0.264^*^	0.095^*^	0.076^*^	0.063^*^
HJ92	0.097	0.188	0.139^*^	0.034	0.014	0.026
LHP80	0.334^*^	0.238^*^	0.250^*^	0.071	0.019	0.040

Asterisks indicate that the fit was significant.

First we note that with respect to standard deviations only P92 and WC99 produce significant fits to the data. When all the data in both experiments are considered, the *R*^2^ value for WC99 and P92 are comparable, interestingly WC99 is rather better than P92 for the 5–200 data in isolation.

Turning to the thresholds we see that WC99 stands out as providing by far the best fit to the data (in terms of variance explained) in both experiments. However, fits for all other models are also significant. Although it does not provide the best fit to the human data, the P92 model has the highest gain (closest to 1) indicating that thresholds for this model are closest in magnitude to those of human observers. It should also be noted that the *R*^2^ value for P92 is low primarily due to the inability of this model to capture human performance when the flow field contains fewer dots.

Overall this analysis suggests that although WC99 provides the best fit to the data and P92 produces thresholds closest in magnitude to the human data, no model can simulate the human data satisfactorily.

**Figure 6:**
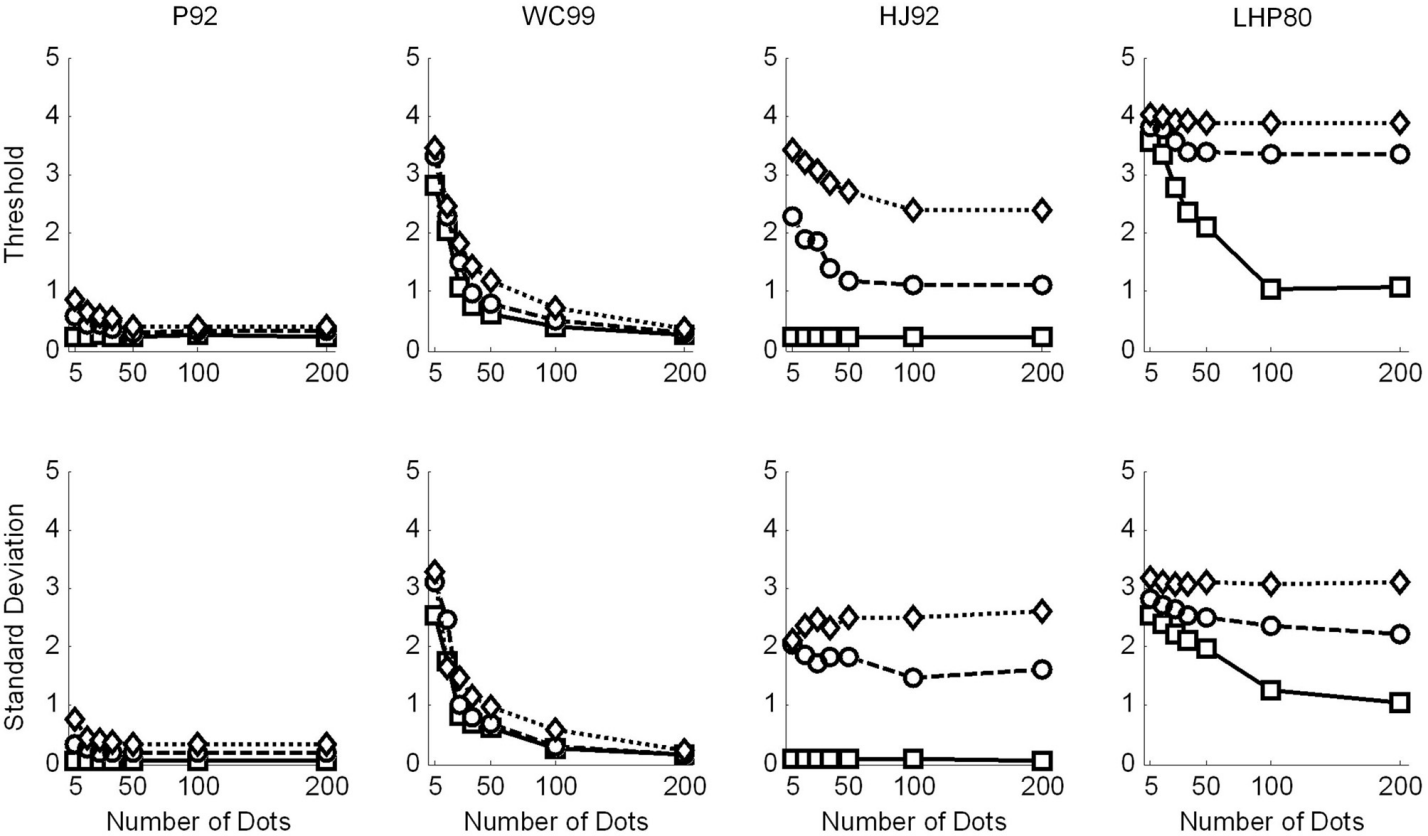
**Raw data for the model observers with a resolution of 0.5°, where appropriate**. Squares are for no noise, circles for noise level 1 and diamonds noise level 2. Top row shows the thresholds recovered in each condition. Simulations terminated when sufficient replications had been conducted to drive the standard error of the mean below 0.2°. Bottom row shows the variability (SD) in the model estimates over 1000 repetitions.

As noted above three models (P92, WC99, HJ92) have an implicit free parameter representing a form of model resolution and this was set to 1° in all three cases. In the case of P92 this parameter controls the resolution of the array of template directions. In the case of WC99 it controls the width of the columns spanning the space of optic flow. For HJ92 there is a parameter which controls the difference between the sampling resolution of the candidate headings. It is possible that the thresholds obtained are critically dependent on the resolution parameter for each model which would impact upon the fits to human data. As a consequence we re-ran all the models (except LHP80) and adjusted the resolution parameter to be 0.5° rather than 1°. The new model simulations are shown in Figure 6. We then repeated the regression analyses and the results are shown in Tables 5 and 6.

**Table 5:** ***R*^2^ statistic for each model for both the thresholds and the standard deviations when the models were rerun with a resolution of 0.5°**.

	**Threshold**			**Standard deviation**		
	**5–50**	**5–200**	**All**	**5–50**	**5–200**	**All**
P92	0.278^*^	0.420^*^	0.343^*^	0.328^*^	0.316	0.303^*^
WC99	0.775^*^	0.825^*^	0.801^*^	0.435^*^	0.872^*^	0.522^*^
HJ92	0.210	0.321	0.272^*^	0.146	0.042	0.092
LHP80	0.561^*^	0.447^*^	0.506^*^	0.119	0.019	0.061

Asterisks indicate that the fitting was significant.

**Table 6:** **Gain parameters (β) for each model for both the thresholds and the standard deviations when the models were rerun with a resolution of 0.5°**.

	**Threshold**			**Standard deviation**		
	**5–50**	**5–200**	**All**	**5–50**	**5–200**	**All**
P92	0.733^*^	1.362^*^	1.077^*^	0.303^*^	0.293	0.306^*^
WC99	0.251^*^	0.285^*^	0.280^*^	0.076^*^	0.084^*^	0.075^*^
HJ92	0.099	0.191	0.142^*^	0.039	0.021	0.030
LHP80	0.334^*^	0.238^*^	0.250^*^	0.071	0.019	0.040

Asterisks indicate that the fitting was significant.

The *R*^2^ values for the three models which were susceptible to resolution effects have remained relatively unchanged suggesting that although this free parameter affects thresholds it cannot change the quality of the fit. WC99 still stands out as providing the best fit to the human data. However, by changing the resolution the impact on the gain parameter is more marked for certain models. In particular, we note that the gains for P92 are now considerably closer to 1 than any other model (they have remained relatively stable for WC99 and HJ90). As a consequence we suggest that it may be possible to tune the performance of certain models (e.g., P92) to produce similar magnitude thresholds to human observers by manipulating the resolution parameter.

## Discussion—model data

In summary the simulations conducted and subsequent analyses suggest that HJ92 and LHP80 are least consistent with human data. In contrast WC99 and P92 do show some similarities to the human data although in different respects. Whilst WC99 provides a better fit to the data overall it appears to exhibit rather higher thresholds than humans. Conversely P92 fits the data less well (primarily due to its inconsistency with human data below 50 dots per frame) but can produce thresholds which are closer in magnitude to those of human observers. Ultimately no model tested provides a good approximation to the thresholds exhibited by humans.

### Improving the models

One anonymous reviewer pointed out that human optic flow processing might be affected by factors such as non-isotropic deployment of attention over the flow field and/or a drop off with increasing eccentricity of the precision of coding motion. Another anonymous reviewer suggested that performance might be improved by whitening the input optic flow (to minimize statistical bias – see e.g., Raudies and Neumann, 2012). The authors of the models we tested did not incorporate such features in their models. We don't know whether that was because they did not consider these issues, whether they did consider them and they judged them irrelevant, or whether they did not consider them relevant to the immediate aim of describing a novel model. In response to the reviewers' comments we decided that we would extend and retest the models in the cases where we felt there was a persuasive biological or empirical basis. Consequently, we conducted further simulations to address eccentricity/precision and the deployment of attention by adding eccentricity dependent noise and changing the spatial configuration of optic flow respectively (these manipulations are explained in Appendix D). We decided not to implement an unbiasing operation because, although there is some suggestion that unbiasing might occur via non-linear response properties of visual neurons (Lyu and Simoncelli, 2009), at least in the case of optic flow processing, there is evidence that human observers do not correct for statistical biases. For example, Hogervorst and Eagle (1998) examined optic flow processing and the recovery of structure from motion and elegantly demonstrated that humans exhibit systematic errors in the estimation of scene structure estimation from optic flow that are not compatible with an unbiasing process.

The results of additional simulations to investigate eccentricity noise scaling and spatial re-configuration of attentional scope to focus on smaller but potentially more informative portions of the scene are described in Appendix D. To summarise neither manipulation could account for the differences between model and human performance and thus our conclusions remain unchanged.

## General discussion

We have investigated the dependence of human heading estimation on the quality and quantity of information in the optic flow field. This was done to provide a reference performance profile for human observers against which we could compare models of optic flow processing to estimate heading.

We found that human performance is sensitive to manipulations in both flow quality and quantity. There appears to be a critical value of flow quantity below 50 dots per frame [possibly in the range of 25–35 dots per frame (Warren et al., 1988)], such that below this point there is a substantial deterioration in performance and above this point performance is relatively stable. With respect to the flow quality we found that human performance degrades as more directional noise is added to the flow field and that this effect is enhanced when the quantity of flow is higher.

Inspection of the threshold profiles suggested that the models approximated the human data with varying degrees of success. The LHP80 model did not exhibit the characteristic dependence on quantity of flow in the noisy conditions. HJ92 appeared to be less robust to noise when compared to human observers. While WC99 thresholds shared some of the characteristics of the human data, it appeared less sensitive to the noise manipulation. Finally although P92 produced the appropriate pattern of thresholds with approximately the correct magnitude it could not account for the data when the flow field was sparse.

Subsequent regression analyses found that LHP80 and HJ92 were least successful in fitting the standard deviation and threshold data. Two models, WC99 and P92, were more successful, although for different reasons. WC99 captured the shape of the human performance profile, which produced the higher *R*^2^ values than P92, but, given an appropriate resolution parameter P92 could produce thresholds which were closer in magnitude to those of human observers.

The fact that LHP80 and HJ92 are least consistent with human data suggests that they are unlikely to provide a good description of the human heading estimation mechanism. However, it should be noted that the motivation behind the design of these models was not to simulate human heading recovery since they represent purely mathematical treatments of the information available in the flow field and an algorithm for recovery of heading.

It is interesting that it was the Bayesian model we tested (WC99) that showed most consistency with human data. This model employs a statistical analysis of the flow field based on simple heuristics about the position of the FoE. In addition, performance of the model also depends upon two other parameters (Appendix B) which are related to the probability of heading direction being between two points in the scene if those points are converging. It may be possible to further tune this model to simulate human performance more closely by adjusting these parameters. At the very least our data should prompt a re-examination of this little-cited model and the principles that underpin it.

Of the two models which did provide better fits to human behaviour one is motivated by human neurophysiology. P92 was based upon the properties of speed and direction tuned cells in MT and the global motion sensors which form templates in this model are at least consistent with the idea of integrating local motion responses from MT in MST. However, as noted, P92 fails to capture the performance of human observers when the flow field is relatively sparse; in particular it performs rather better than humans in such circumstances. Therefore P92 would need to be extended or modified to better capture human performance with sparse flow fields.

## Conclusions

In conclusion we suggest that of the four models tested two stand out as most consistent with human data. One of these models is based on a heuristic analysis of the flow field combined with a Bayesian combination of available evidence for heading direction. The other is based upon properties of cells in neural motion processing regions. We note however, that none of the models considered is able to fully capture human heading performance in response to manipulation of the quantity and quality of information in the flow field.

### Conflict of interest statement

The authors declare that the research was conducted in the absence of any commercial or financial relationships that could be construed as a potential conflict of interest.

## Figures and Tables

**Figure B1:**
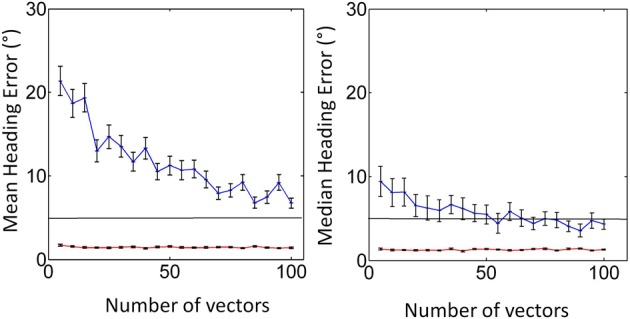
**Replication of Figure 5 from P92 using mean error (left) and median error (right)**. Shown are the cases for no noise (red), and aperture noise (blue). Error bars represent ±1 standard error.

**Figure B2:**
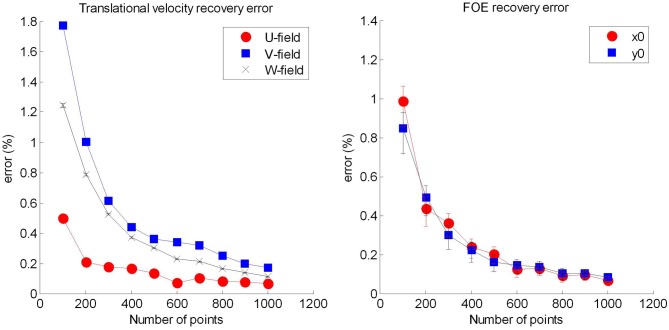
**Testing the LHP80 algorithm**. (Left) recovery of the translational velocity vector showing all three components, and (right) the recovery of the focus of expansion (vanishing point). Error bars represent ±1 standard error.

**Figure B3:**
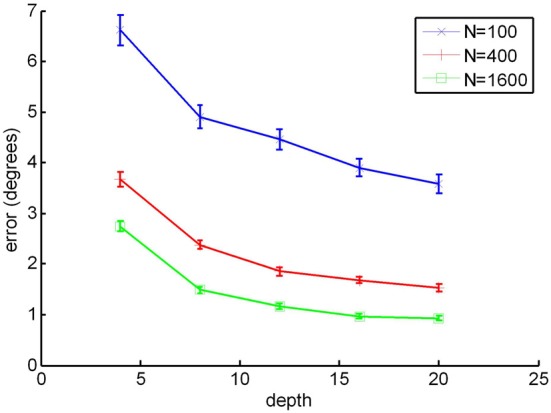
**Recreation of the Figure 5 from WC99, where N is the number of vectors in the flow field**. Error bars represent ±1 standard error.

**Figure C1:**
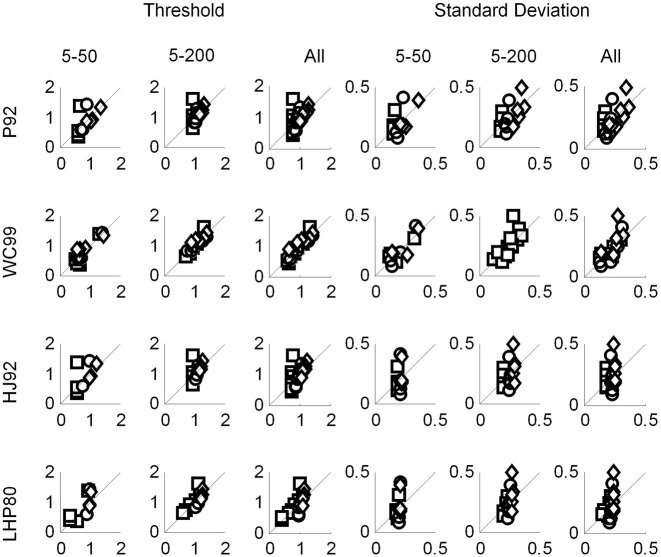
**Regression plots with the human data on the vertical axis and the predicted data on the horizontal axis**. The data for P92, WC99 and HJ92 were generated with a resolution of 1°.

**Figure C2:**
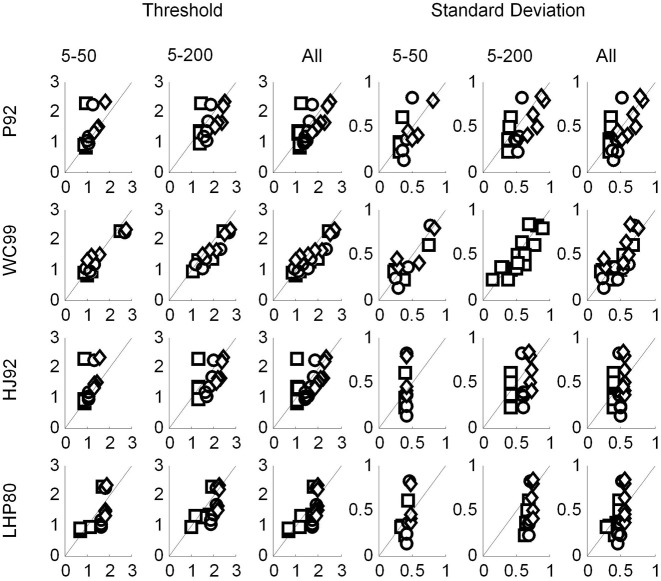
**Regression plots with the human data on the vertical axis and the predicted data on the horizontal axis**. The data for P92, WC99 and HJ92 were generated with a resolution of 0.5°.

**Figure D1:**
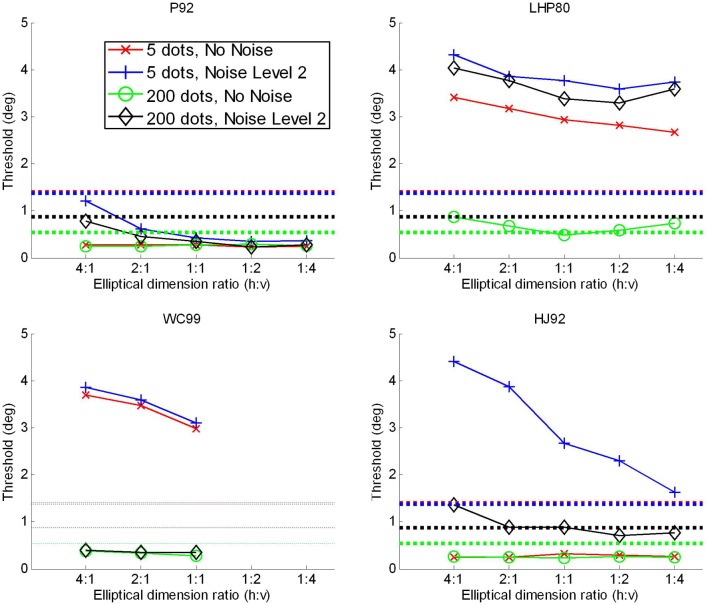
**Attentional scaling**. Results of additional simulations for all four models.

**Figure D2:**
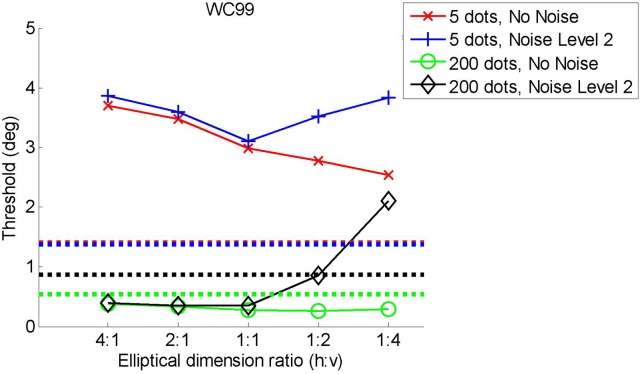
**Attentional scaling for WC99 with a larger standard area**.

**Figure D3:**
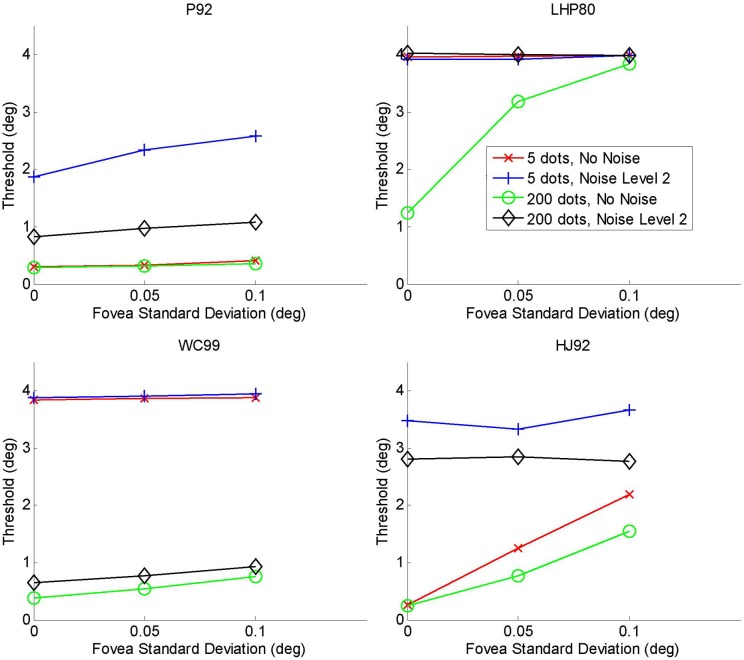
**Eccentricity scaling plots for the four model observers**.

## Appendices

### Appendix A: weighting of data based on goodness of fit

Once thresholds were normalised, the overall mean for the experiment was calculated using weights for each individual. Firstly, the root mean square of the error of fitting for the 12 psychometric curves for each observer was calculated. This was taken as the difference between the actual data point and the fitted data point, squared and added to the other squared errors before square rooting the result. Once the RMS's had been calculated, the weights were calculated as

$$w_{n,d}^{i}=\frac{\frac{1}{r_{n,d}^{i}}}{\sum_{i=1}^{N}\frac{1}{r_{n,d}^{i}}}$$

where *r*_*i*_ = RMS for observer *i* at condition noise, *n*, and number of dots, *d*. Then the mean for each noise (*n*) + number of dots (*d*) condition over *N* observers is given by

$$m_{n,d}=\sum_{i=1}^{N}w_{n,d}^{i}m_{n,d}^{i}$$

where *m*^*i*^_*n*, *d*_ is the mean for observer *i* at conditions noise, *n*, and number of dots, *d*. Strictly we should have that *m*_*n*, *d*_ is divided by the sum of the weights. But since $\sum_{i=1}^{N}w_{i}=1$, then there is no need to included this. This therefore gave a fair and balanced view of the data where in some instances the fitting of the psychometric curves was maybe not that good.

The standard errors were calculated in a similar manner and are given by the formula

$$s_{n,d}=\sqrt{\frac{\sum_{i=1}^{N}w_{i}{\left(m_{n,d}^{i}-m_{n,d}\right)}^{2}}{N}}$$

such that *s*_*n*, *d*_ is the standard error for the noise condition *n* with number of dots *d*.

### Appendix B: model implementation

#### P92

Perrone (1992) uses a Gaussian to represent activity in both directional and speed tuned sensors. The directional tuned output function is then given by

$$O_{d}(x)=\frac{e^{\left(-0.5{\left(\frac{x}{30}\right)}^{2}\right)}-\delta }{1-\delta }$$

where *x* is the difference, in degrees between the preferred direction of motion of the sensor and the direction of the image motion, and δ is a parameter determining the amount of inhibitory output from the unit. For this study, as in P92, δ = 0.05 for all simulations. The preferred direction of the sensor is the direction from the sensor location (α, β) to the image location. If the sensor was located at the FOE, we could see that the direction of the image motion and the preferred direction of the sensor would be the same, giving us a maximum value of the output function *O*_*d*_.

The speed tuned sensor's output function is given by

$$O_{s}(y)=e^{\left(-0.5y^{2}\right)}$$

where, given the actual image speed *r* and the optimum speed for the sensor *R*, $y=\log_{2}\frac{r}{R}$. If *r* = *R*, then we see that $O_{s}\left(\log_{2}\frac{R}{R}\right)=1$, giving us the maximum output of the sensor when the image speed is the same as the sensor speed.

Once the directional and speed outputs have been calculated, then the overall detector output is given by

$$O_{\alpha ,\beta }=\sum_{j=\;0}^{m}\max{\left\{O_{d}(\theta_{j}-\Xi_{j})O_{s}\left[\log_{2}\left(\frac{r_{j}}{R_{j,k}}\right)\right]\right\}}_{k=1}^{n}$$

where *m* is the total number of motion sensors responding to the image motion presented by the image field, i.e., the number of image points in the plane, and *n* is the number of speed tuned motion sensors in each detector. Throughout our simulations, we fixed the number of speed tuned sensors at *n* = 4.

Our implementation of this model was tested by attempting to recreate Figure 5 from Perrone (1992). In this simulation heading recovery was tested as a function of the number of elements in the flow field and in the presence of noise designed to simulate the aperture problem. We show our results in Figure B1 which are similar to those presented in Perrone (1992).

#### LHP80

This model first recovers the “vanishing point” (VP) for the flow field, which is in essence the FOE; then from this the rotational velocities can be recovered, followed by the relative depths and lastly the translational velocities.

For an observer moving with translational velocity (*U*, *V*, *W*) and rotational velocity (*A*, *B*, *C*), the velocity (*u*, *v*) of a point at location (*x*, *y*) in the image plane is given by

$$\begin{array}{l} u=\left(-\frac{U}{Z}-B+Cy\right)-x\left(-\frac{W}{Z}-Ay+Bx\right),\\ v=\left(-\frac{V}{Z}-Cx+A\right)-y\left(-\frac{W}{Z}-Ay+Bx\right). \end{array}$$

The LHP algorithm takes advantage of the fact that image velocities can be decomposed into translational and rotation components:

$$\begin{array}{l} u^{T}=\frac{\left(-U+xW\right)}{Z},v^{T}=\frac{\left(-V+yW\right)}{Z},\\ u^{R}=-B+Cy+Axy-Bx^{2},v^{R}=-Cx+A+Ay^{2}-Bxy. \end{array}$$

The VP (*x*_0_, *y*_0_) is directly related to the translational velocities and its coordinates are given by

$$x_{0}=\frac{U}{W},y_{0}=\frac{V}{W}.$$

The VP can be recovered by solving the translational velocity equation for *U*, *V*, *W*. In order to do this there must be 2 pairs of unique image objects such that each pair lie on the same rays from the eye, i.e., each pair of image points lie at the same point on the image plane but at different depths. The intersection of these two rays is the VP.

As noted, the recovery of the VP depends on there being two pairs of points at the same location in the image but at different depths. In sparse flow fields this is rather unlikely. If, however, there are such points, then the exact position of the VP can be recovered.

Unfortunately, in the original paper the authors did not present any data for us to check our implementation against. However, we conducted our own test.

Keeping the rotational and translational velocities fixed [(0.1,0.1,0.1) and (0,0,0) respectively], we ran the code for 10,000 simulations, and calculated the error of generating the translational velocity (*U*, *V*, and *W* values), and the FOE.

We show the results in Figure B2.

We see that for the recovery of the *U*, *V*, *W* values, the errors for the *V* and *W* values are much higher than the errors for the *U* value. The reason for this is that the algorithm implemented is such that the *U* value is calculated first. This value is then used to calculate *V* and *W*. Therefore, the error in the *U* calculation is magnified when calculating the *V* and *W* values.

#### HJ92

The starting point for this model is the equation of the optic flow field, ν(*x*, *y*) for an image point with image plane coordinates (*x*, *y*) (this is a restatement of the pair of equations provided in the LHP80 models)

$$\nu (x,y)=p^{\prime }(x,y)A(x,y)T^{\prime }+B(x,y)\Omega$$

where *p*′(*x*, *y*) = 1/*Z* are the inverse depths, ***T***′ & Ω are the translational and rotational velocities respectively. The matrices ***A***(*x*, *y*) & ***B***(*x*, *y*) are given by

$$\begin{array}{l} A(x,y)=\left[\begin{array}{ccc} -f & 0 & x\\ 0 & -f & y \end{array}\right],\\ B(x,y)=\left[\begin{array}{ccc} \frac{xy}{f} & -\left(f+\frac{x^{2}}{f}\right) & y\\ f+\frac{y^{2}}{f} & -\frac{xy}{f} & -x \end{array}\right]. \end{array}$$

where *f* is the focal length.

We note that both *p*′(*x*, *y*) and ***T***′ are unknowns and therefore, since they are multiplied, they can only be determined up to a scale factor. This implies that we can only solve for the direction of the translation (not the magnitude) and relative (not absolute) depth. Consequently Heeger and Jepson (1992) reformulate the equations to use the relative depth, *p* = ∥*T*′∥/*Z*, and a unit vector in the translational direction, ***T***.

For a flow field that has *N* sample points, the optic flow equations stated above can be rewritten as

$$\begin{array}{l} v=A(T)p+B\Omega ,\\ \;=C(T)q, \end{array}$$

where ***v*** is a **2***N* vector of image velocities, ***p*** now a *N*-vector containing the inverse depths at each sample point and ***A***(***T***) is a **2***N* × *N* matrix

$$A(T)=\left[\begin{array}{ccc} A(x_{1},y_{1})T & \cdots & 0\\ \vdots & \ddots & \vdots \\ 0 & \cdots & A(x_{N},y_{N})T \end{array}\right]$$

Similarly, ***B*** is a **2***N* × 3 matrix and is given by

$$B=\left[\begin{array}{c} B(x_{1},y_{1})\\ \vdots \\ B(x_{N},y_{N}) \end{array}\right]$$

Finally, ***q*** is an *N* + 3 vector obtained by collecting the unknown depth and rotation components, and ***C***(***T***) is obtained by concatenating ***A*(*T*)** and ***B***

$$C(T)=\left[\begin{array}{cc} | & |\\ A(T) & B\\ | & | \end{array}\right].$$

The HJ92 algorithm is based on a numerical approach in which the best estimate of the unknowns (***T***, Ω, ***p***) is obtained by minimising the residual function

$$E\left(T,q\right)=\|v-C(T)q{\|}^{2}.$$

However, since this minimisiation proves to be computationally expensive the residual is reformulated as

$$E(T)=\|v^{t}C^{⊥}(T){\|}^{2},$$

where ***C***^⊥^ is the orthogonal complement of the matrix ***C***. As can be seen, this residual function is now just dependent on the unit translational velocities ***T***.

In addition, the algorithm splits the visual space into discrete regions and samples each region separately for candidate translational directions. The residuals for each region are then added to together to get a result for the complete space.

Once the translational velocities have been recovered, the rotational velocities can also be recovered by eliminating the depths *p*′(*x*, *y*) from the optic flow equation. Heeger and Jepson do this by introducing a unit vector perpendicular to the translational component of the flow field and multiplying both sides of the original equation by this new vector to eliminate the depth. This equation can then be solved for Ω, and the depths can be recovered by minimising the residual

$$E(\Omega )=\sum_{i}\|d_{i}^{t}B_{i}\Omega -d_{i}^{t}v_{i}{\|}^{2},$$

where ***d***^*t*^_*i*_ is the new vector introduced above which is perpendicular to the translational component of the flow field.

Finally, once the rotation and translation velocities have been determined, the optic flow equation can once again be used to find the depths.

For heading, we only need to recover the translation velocities, which eases the computational effort somewhat. However, depending on the size of the image sample, *N*, and also the number of regions to separately sample, this method can be computational expensive.

As a test that our algorithm was a faithful replication, we took Figure 5 from HJ92 and ran the code for the conditions used in that figure for no noise (0.0) and when noise was 0.2. We found that our values of the error were 0.4 (approximately 0 in the original figure) for no noise and 12.4 for the 0.2 noise condition (approximately 12 in the original figure). Therefore, we are confident that our algorithm is implemented correctly.

### WC99

Wang and Cutting (1999) start by considering the angular velocity of a particular point on the image plane. Assuming that the only rotation of the eye possible is around the *y*-axis and that the field of view is “reasonably small”, the following theorem can be shown to hold:

Theorem 1: *If, during camera motion, the projections of two stationary points I′*(θ_*i*_, ϕ_*i*_) *and J′*(θ_*j*_, ϕ_*j*_) *move closer to each other, e.g., in the X direction (i.e.,* (θ_*i*_ − θ_*j*_)*d*θ_*ij*_/*dt* < 0), *then the direction of camera translation can be to either side of the two points but not in between them (i.e.,* α > max{θ_*i*_, θ_*j*_} or α < min{θ_*i*_, θ_*j*_}).

where *d*θ_*ij*_/*dt* is the relative angular velocity between two points *i* and *j* given by

$$d\theta_{ij}/dt=d\theta_{i}/dt-d\theta_{j}/dt$$

and

$$d\theta_{i}/dt=\frac{x_{i}V_{z}-z_{i}V_{x}}{x_{i}^{2}+z_{i}^{2}}+\frac{x_{i}y_{i}\omega_{x}}{x_{i}^{2}+z_{i}^{2}}-\omega_{y}+\frac{y_{i}z_{i}\omega_{z}}{x_{i}^{2}+z_{i}^{2}}$$

where the translational velocity is given by (*V*_*x*_, *V*_*y*_, *V*_*z*_) and the rotational velocity is given by (ω_*x*_, ω_*y*_, ω_*z*_) in the *x*, *y*, *z* directions. The coordinate of a point in the optic flow field is given by (*x*, *y*, *z*).

Now, let *p*(*x*) be the probability of the camera heading towards position *x*. Also, let *C*_*ij*_ = 1 if two points at positions *i* and *j* form a converging pair, and *C*_*ij*_ = 0 if they do not. The conditional probability of the two points converging (*C*_*ij*_ = 1) or not converging (*C*_*ij*_ = 0) given the heading aimpoint *x*, *p*(*C*_*ij*_/*x*), is defined as

$$\begin{array}{l} p(C_{ij}=1/x)=\{\begin{array}{c} \varepsilon \text{ if }i<x<j\\ \eta \text{ if }x<i\text{ or }x>j \end{array}\\ p(C_{ij}=0/x)=\{\begin{array}{c} 1-\varepsilon \text{ if }i<x<j\\ 1-\eta \text{ if }x<i\text{ or }x>j \end{array} \end{array}$$

where ε and η are fixed parameters. In practice, ε is a small value since the aimpoint cannot lie between two converging points, according to the theorem stated above. However, when the flow field is noisy, Wang and Cutting (1999) suggests that the parameter should be increased accordingly. Therefore, for our simulations we used the default value of ε to be 0.01 if there was no noise, and 0.2 and 0.4 for when there was noise for noise levels 1 and 2 respectively. Also, η parameter should be large since two points in the flow field on the same side of the heading point may or may not be converging, again according to the above theorem. Hence we use η = 0.5, as was used in Wang and Cutting (1999).

Using Bayes' rule we get *p*(*x*/*C*_*ij*_) = *p*(*C*_*ij*_/*x*)*p*(*x*). Therefore, if two points at positions *i* and *j* are converging, then the probability of them heading in direction *x* is given by

$$\begin{array}{l} p(x/C_{ij}=1)=\varepsilon p(x),\text{ if }i<x<j\\ p(x/C_{ij}=1)=\eta p(x),\text{ if }x<i\text{ or }x>j \end{array}$$

or, if they do not converge

$$\begin{array}{l} p(x/C_{ij}=0)=(1-\varepsilon )p(x),\text{ if }i<x<j\\ p(x/C_{ij}=0)=(1-\eta )p(x),\text{ if }x<i\text{ or }x>j \end{array}$$

In WC99, pairs of points are sequentially sampled and the posterior probability, *p*(*x*/*C*_*ij*_), for heading direction given the presence or absence of convergence is calculated. Crucially, at each step the newly calculated posterior becomes the prior probability for the next pair, *p*(*x*). Once the probabilities for all pairs have been calculated the maximum normalised posterior probability is used to recover heading direction.

Wang and Cutting (1999) describes an algorithm which incorporates this theorem to provide a posterior for heading direction. However, the algorithm does not sample and assess every single pair of points for convergence since this would be computationally expensive for dense flow fields. In order to get around this the field is split into columns and rows, and the above method is applied on a column and row basis (where columns and rows are 1 degree wide).

Again, as with the other models, we replicated one of the figures presented in the original paper to show that our algorithm was implemented correctly. This time, we took Figure 3 from Wang and Cutting (1999), and show our results in Figure B3. As in Wang and Cutting (1999) we manipulated the number of points and depth.

We see that on comparison to Figure 3 from the original paper, there errors generated for the 400 and 1600 dot conditions are very similar. For the 100 dot condition the first two points (depths 4 and 8) appear to be approximately 27% and 8% larger respectively than those shown the original paper. This may be due to discrepancies in the epsilon and eta parameters. However, given that the majority of data points appear to be close to those reported in WC99, we feel confident that we have correctly implemented this model.

### Appendix C: regression analysis

In this appendix we provide more details of the regression analysis that was performed to how well the model data fitted to the human data.

As described in the main body of this article, we performed linear regression analyses on the human and the model data in order to establish which model, if any, resembled the human data. We performed two separate analyses. The first was with the model data generated using a 1° resolution (Figure C1), and the second using a 0.5° resolution (Figure C2).

### Appendix D: effects of non-isotropic attention and eccentricity scaling

Human vision is subject to cognitive and perceptual artefacts that are not taken account of in the models tested. An anonymous reviewer suggested that we consider two in particular: (1) changes in attention scope (e.g., the tendency in certain circumstances for attention to be narrowed over a particularly salient or informative portion of the scene – e.g., horizontal or vertical meridians rather than focussed in a circular region); and (2) eccentricity scaling (i.e., the tendency for precision to decrease as a function of retinal eccentricity). Here, we discuss the effects of these factors on our results and show that they do not interfere with the major conclusions of this article.

#### Effects of non-isotropic attention

It has been suggested that when making judgements of horizontal heading direction, flow vectors close to the vertical meridian are more useful than those close to the horizontal meridian (van den Berg, 1996). Consequently, human observers might actively deploy attention on this particularly salient region of space.

We tested the effects of by changing the shape of the window in which motion was presented by restricting the flow field to an elliptical image region. We initially conducted simulations in which all flow vectors were restricted to appear in a circular window of radius 5 degrees. In order to allow a fair comparison, we then generated elliptical windows for the flow with the same area as the circle (25*p* deg^2^). The radii were chosen so that the horizontal:vertical ratios were 4:1, 2:1, 1:1, 1:2, 1:4. We tested the extremities of the parameter ranges from the main experiment presented above, i.e., the 5 and 200 dots per frame conditions and the no noise and noise level 2 conditions. Note, however, that in these simulations the effective density is considerably higher than in the main experiment, since, for example, the 200 dots are now all present in a region of the field with considerably smaller area than the full screen which was used in the main experiment. Consequently direct comparison between this simulation and that above is not possible. The results of the simulations are shown in Figure D1.

Considering the models in turn: P92 shows worse performance as the flow field gets wider and shorter. Performance was constant from 1:1 (round) to 1:4 (thin/high). For noisy or sparse flow fields LHP80 shows an improvement in performance from 4:1 through to 1:4. As with P92, WC99 shows a drop-off in performance as the flow field becomes wider and shorter. As the flow field becomes thinner and taller WC99 no longer produces an estimate—this is because a fundamental requirement (that the flow field contains the FoE) is no longer fulfilled. We have run a 2nd simulation with this model in which we (trebled) the area with the results shown in Figure D2. Once we have done that the FoE remains within the flow field and now we find that performance decreases as the ratio changes from 1:1 to 1:2 and 1:4 for noise level 2. HJ92 shows relatively stable performance apart from in the noise level 2 conditions where performance increases as the flow field becomes thinner and taller. In summary, we find no results here that would make us change our interpretation of the main experiments.

#### Eccentricity scaling

All visual features, including motion, are coded with decreasing precision in the periphery. In the case of heading perception it has also been shown (Crowell and Banks, 1993) that heading recovery thresholds increase with the retinal eccentricity of the flow field (although these effects are rather small over the range of eccentricities tested here). We simulated such effects by adding Gaussian noise with a standard deviation that was proportional to the log polar distance of the point from the centre of the flow field.

We implemented the noise model as a shift in the coordinates of the flow field dots in consecutive frames. The shift was sampled from a zero mean Gaussian distribution. Consider a dot at position (*x*_0_, *y*_0_). It's new position is shifted to (*x*′, *y*′), where *x*′, is given by

(1) $$x^{\prime }=x_{0}+N(0,\sigma (\ln(\varepsilon ))),$$

and *y*′ has the same form as D1. The variable σ(ln(ε)) is the standard deviation dependent on the log of eccentricity, ε.

All four models were tested and we show the results in Figure D3. We looked at the extremities of the parameter ranges from the simulations conducted in the main text, i.e., the 5 and 200 dots per frame conditions and the no noise and noise level 2 conditions. We show results for three different values of the fovea s.d., one of which is zero which corresponds to no eccentricity noise scaling and is therefore comparable to the simulations presented in the main analysis.

In all cases, the addition of further eccentricity dependent noise has made the models somewhat worse. LHP80 performs poorly which is consistent with our findings above that it is particularly sensitive to noise. The noise manipulation made very little difference to WC99 and also to P92. For the maximum noise condition, there was little change in performance for HJ92, but for no noise we see that as we increase the noise due to eccentricity scaling, performance decreases rather markedly.

Overall, Figure D3 suggests that eccentricity scaling leads to an overall deterioration in performance and this is particularly marked for the LHP and HJ models. Interestingly the two models singled out in our discussion as having most in common with human performance show some robustness to the eccentricity scaling manipulation. We see nothing in these results which would make us change our conclusions.
